# Interaction of irritability and anxiety on emotional responding and emotion regulation: a functional MRI study

**DOI:** 10.1017/S0033291720001397

**Published:** 2021-12

**Authors:** Kathleen I. Crum, Soonjo Hwang, Karina S. Blair, Joseph M. Aloi, Harma Meffert, Stuart F. White, Patrick M. Tyler, Ellen Leibenluft, Kayla Pope, R. J. R. Blair

**Affiliations:** 1 Medical University of South Carolina, Charleston, South Carolina, USA; 2 Center for Neurobehavioral Research, Boys Town National Research Hospital, Boys Town, Nebraska, USA; 3 University of Nebraska Medical Center, Omaha, Nebraska, USA; 4 Target Holding, Groningen, The Netherlands; 5 Emotion and Development Branch, National Institute of Mental Health, Bethesda, Maryland, USA; 6 Medical College of Wisconsin, Northeastern Wisconsin Psychiatry Training Program, Winnebago, Wisconsin, USA

**Keywords:** Anterior cingulate cortex, anxiety, irritability, rostro-medial prefrontal cortex

## Abstract

**Background:**

Irritability and anxiety frequently co-occur in pediatric populations. Studies separately looking at the neural correlates of these symptoms have identified engagement of similar neural systems – particularly those implicated in emotional processing. Both irritability and anxiety can be considered negative valence emotional states that might relate to emotion *dysregulation*. However, previous work has not examined the neural responding during the performance of an emotion regulation task as a function of interaction between irritability and anxiety simultaneously.

**Methods:**

This fMRI study involved 155 participants (90 with significant psychopathologies and 92 male) who performed the Affective Stroop Task, designed to engage emotion regulation as a function of task demands. The Affective Reactivity Index (ARI) was used to index irritability and the Screen for Child Anxiety Related Emotional Disorders (SCARED) was used to index anxiety.

**Results:**

Levels of irritability, but not anxiety, was positively correlated with responses to visual images within the right rostro-medial prefrontal cortex and left anterior cingulate cortex during view trials. The second region of ventral anterior cingulate cortex showed a condition-by-emotion-by-ARI score-by-SCARED score interaction. Specifically, anxiety level was significantly correlated with a decreased differential BOLD response to negative relative to neutral view trials *but only in the presence of relatively high irritability*.

**Conclusions:**

Atypical maintenance of emotional stimuli within the rostro-medial prefrontal cortex may exacerbate the difficulties faced by adolescents with irritability. Moreover, increased anxiety combined with significant irritability may disrupt an automatic emotional conflict-based form of emotion regulation that is particularly associated with the ventral anterior cingulate cortex.

## Introduction

Irritability is defined as an ‘increased propensity to exhibit anger relative to one's peers’ (Leibenluft, 2017, p.277) and a ‘relative dispositional tendency to respond with anger to blocked goal attainment, and includes both mood (trait) and behavioral (reactive state) dysregulation’ (Camacho, Karim, & Perlman, 2019; Fishburn et al., 2019, p.69; Stringaris et al., 2012; Wakschlag et al., 2018). It is the hallmark symptom of the Diagnostic and Statistical Manual of Mental Disorders version 5 (DSM-5) criteria for Disruptive Mood Dysregulation Disorder (DMDD) (American Psychiatric Association, 2013). Moreover, it is a symptom prevalent in many other psychiatric diagnoses including major depressive disorder (MDD) (Stringaris, Maughan, Copeland, Costello, & Angold, 2013), bipolar disorder (Berk et al., 2017), attention-deficit/hyperactivity disorder (Karalunas et al., 2014), and oppositional defiant disorder/conduct disorder (Young et al., 2009). Maladaptive irritability is one of the most common reasons children present to a clinic for mental health services (Avenevoli, Blader, & Leibenluft, 2015).

While increased irritability is seen in many patient groups, a specific symptom that frequently co-occurs with irritability in both clinical and community samples is anxiety (Cornacchio, Crum, Coxe, Pincus, & Comer, 2016; Kircanski et al., 2018; Stoddard et al., 2014). Indeed, youths with significant levels of irritability are at an increased risk for the future development of anxiety disorders (Savage et al., 2015; Stringaris & Goodman, 2009). Moreover, models of the pathophysiology underpinning irritability and anxiety show considerable overlap. Heightened responsiveness to threat signals within the amygdala and connected neural structures has been related to both irritability (Blair, 2018; Kircanski et al., 2018; Leibenluft, 2017; Wiggins et al., 2016) and anxiety (Blair et al., 2008; LeDoux & Pine, 2016; Salum, Desousa, do Rosario, Pine, & Manfro, 2013).

Despite this high comorbidity and apparent overlap in pathophysiology, the stark differences in symptom manifestation suggest that there must be distinctions between these two psychopathologies at the neurobiological level. Up to the present, two studies have examined this question (Kircanski et al., 2018; Stoddard et al., 2017). One, using a facial expression task, reported that when responding to extreme anger expressions (150% intensity morphs), high levels of both irritability *and* anxiety were associated with *reduced* amygdala–medial prefrontal cortex (mPFC) connectivity, whereas high levels of anxiety and *low* levels of irritability were associated with *increased* amygdala–mPFC connectivity (Stoddard et al., 2017). The second study, using a facial expression dot probe task, reported a *positive* correlation between irritability and BOLD responses to threat-incongruent relative to threat-congruent trials within the dorsolateral, ventrolateral prefrontal and insula cortices while there was a *negative* correlation between levels of anxiety and amygdala–cingulate connectivity (Kircanski et al., 2018).

Both irritability and anxiety can be considered as forms of emotion *dysregulation* (Blair et al., 2012; Leibenluft & Stoddard, 2013). Emotion regulation is a broad term that includes various control processes (Gyurak, Gross, & Etkin, 2011; Ochsner & Gross, 2005; Phillips, Drevets, Rauch, & Lane, 2003): One involves prefrontal (dorsomedial, lateral frontal and anterior cingulate cortices) and parietal cortices in a top-down attention-driven process (Buhle et al., 2014; Ochsner & Gross, 2005). A second is thought to implicate ventromedial prefrontal cortex (vmPFC)/ventral anterior cingulate cortex in an emotional conflict process (Gyurak et al., 2011). Data have suggested that deficient emotion regulation in anxiety might relate to both deficient top-down attentional processes (Blair et al., 2012) and/or down regulation via vmPFC (Etkin & Wager, 2007). But previous work has not examined any potential differential functional integrity of either of these emotion *regulation* mechanisms with respect to irritability and anxiety. While differential correlational patterns between amygdala and mPFC might reflect differences in forms of emotion dysregulation with respect to irritability and anxiety (Stoddard et al., 2017), this study did not employ a neurocognitive task designed to recruit emotion *regulation*. The current study aims to address this gap in the literature.

**Fig. 1.**
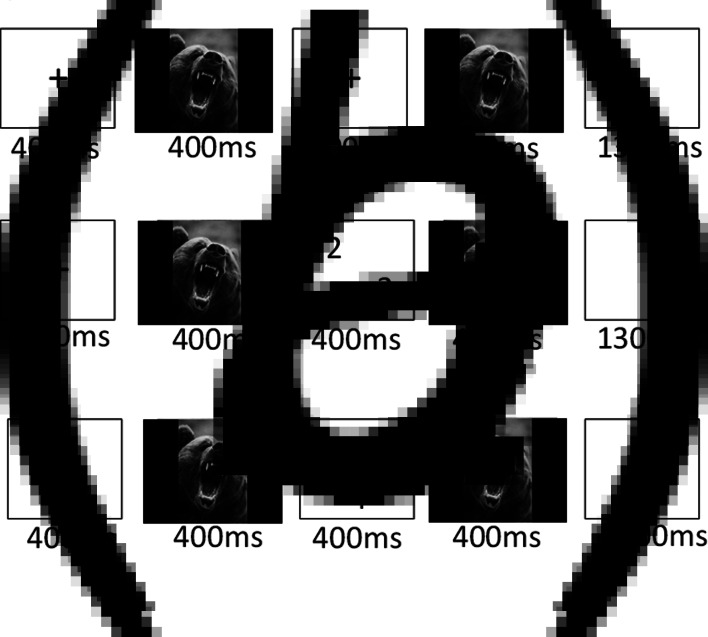
In this task, the participant is asked to count how many numbers are presented on the screen. Numbers may match the value of the number (congruent, *b*) or not (incongruent, *c*). The numbers are bracketed between emotional stimuli (negative, positive, neutral). The participants also view the emotional stimuli pictures without counting numbers (view, *a*). There are two runs, with 48 trials for each emotional stimuli.

In this study, we implemented an emotion distraction task, the Affective Stroop Task (AST) (Blair et al., 2007; Hwang et al., 2016) to a relatively large cohort of youths [*n* = 155, including youth from both the community (*n* = 65) and a residential treatment program for adolescents with internalizing and externalizing conditions (*n* = 90); for details, see below]. In this task, participants either view the emotional/neutral images (Fig. 1*a*) or perform goal-directed activity (counting the number of numerals) in the context of emotional and neutral distracter images (Fig. 1*b* and *c*). As such, the task indexes systems responsive to: (i) emotional stimuli (amygdala and mPFC: main effect of emotion); (ii) undisturbed visual stimuli (amygdala and mPFC: main effect of condition: view>congruent/incongruent); (iii) goal-directed functioning (dorsomedial and lateral frontal, anterior insula, parietal, premotor, and motor cortices; main effect of condition: congruent/incongruent>view); and (iv) emotion regulation [amygdala; emotion-by-condition interaction: view(positive/negative-neutral)>congruent/incongruent(positive/negative-neutral)] (e.g. Blair et al., 2007; Blair et al., 2013; Hwang et al., 2016). For a conceptual overview of this task, see online Supplementary Fig. S1. Notably, within this task, the response to visual stimuli during view trials within the amygdala and vmPFC is typically at least as strong as the response to emotional stimuli (see online Supplementary Fig. S2).

We had three hypotheses. H1: Patients with irritability and/or anxiety show amygdala and mPFC hyper-responsiveness (see Hur, Stockbridge, Fox, & Shackman, 2019; LeDoux & Pine, 2016; Leibenluft, 2017). The amygdala and mPFC show differential responsiveness on the AST to: (i) emotional relative to neutral trials; and (ii) view relative to congruent/incongruent trials; see online Supplementary Fig. S2. As such, increased responsiveness within the amygdala and mPFC, as a function of irritability and/or anxiety, might manifest in two possible ways; i.e. as a greater difference in responsiveness between: (i) positive/negative and neutral trials (irritability and/or anxiety should be positively associated with responsiveness during negative/positive trials; H1A); and/or (ii) view and congruent/incongruent trials (irritability and/or anxiety should be positively associated with responsiveness during view trials; H1B). H2: Following previous work with this task with patients with anxiety disorders (Blair et al., 2012; Blair et al., 2013; Roy, Costanzo, Blair, & Rizzo, 2014; White et al., 2018; White, Costanzo, Blair, & Roy, 2015), we predicted that increased levels of anxiety would be associated with reduced recruitment of regions implicated in goal-directed functioning (e.g. dorsomedial frontal, lateral frontal and parietal cortices) during goal-directed trials (congruent and incongruent) relative to view trials. H3: Increased levels of irritability and/or anxiety would relate to decreased recruitment of regions implicated in emotion regulation (showing in the condition-by-emotion interaction).

## Methods

### Participants

One hundred and fifty-five participants (92 male), aged 10–18, were recruited from a residential treatment program for behavioral and emotional problems [Boys Town; *n* = 90; *n*(males) = 54] and the surrounding local community [*n* = 65; *n*(males) = 38]. Study participants consisted of healthy volunteers [*n* = 57; *n*(males) = 32] and youths with internalizing and externalizing conditions [*n* = 98; *n*(males) = 60]; for demographic and clinical details (see Table 1). Clinical assessment/characterization was done through psychiatric interviews with the participants and their parents by licensed and board-certified child and adolescent psychiatrists who adhered closely to common clinical practice. The institutional review board of Boys Town National Research Hospital approved the study. A doctoral-level researcher or a member of the clinical research team obtained written informed consent and assent. In all cases, children and adolescents had the right to decline participation at any time before or during the study. Consent documents were reviewed with parents/legal guardians and written permission was obtained (1) at the initial visit for community participants or (2) at the time of intake for children and adolescents placed in Boys Town treatment programs. Assent was obtained from the Boys Town youths in a separate session but only after parental consent.

**Table 1.** Demographics

	Total participants (*n* = 155) Mean (s.d.)	Correlations with ARI	Correlation with SCARED
Age	15.30 (2.17)	0.142	−0.014
Sex	92/63 M/F	−0.034	−0.309*
IQ	102.89 (11.63)	−0.228*	0.218*
ARI scores	2.68 (2.84)	–	0.429*
SCARED scores	19.46 (14.91)	0.429*	–
Diagnosis			
ADHD	60 (38.71%)	0.278*	0.141
ODD	63 (40.65%)	0.339*	0.121
CD	51 (32.90%)	0.245*	0.112
MDD	22 (14.19%)	0.347*	0.473*
GAD	38 (24.52%)	0.404*	0.607*
SAD	29 (18.71%)	0.335*	0.641*
PTSD	18 (11.61%)	0.298*	0.466*
Medications			
Stimulants	15	0.215*	0.207*
SSRIs	16	0.136	0.152
Anti-psychotics	6	0.165*	0.030

s.d., standard deviation; IQ, intelligence quotient; ARI, Affective Reactivity Index; SCARED, Screen for Child Anxiety Related Disorders. No significant differences were observed between males and females on age, IQ, ARI, and SCARED scores; ADHD, attention-deficit/hyperactivity disorder; ODD, oppositional defiant disorder; CD, conduct disorder; MDD, major depressive disorder; GAD, generalized anxiety disorder; SAD, social anxiety disorder; PTSD, post-traumatic stress disorder; SSRIs, selective serotonin reuptake inhibitors; *p < 0.05.

IQ was assessed with the Wechsler Abbreviated Scale of Intelligence (two-subtest form; Wechsler, 1999). Parents as well as children/adolescents completed the Affective Reactivity Index (ARI) (Stringaris et al., 2012), a scale measuring irritability with demonstrated reliability and validity (Stoddard et al., 2014). Children and adolescents completed the Screen for Child Anxiety Related Emotional Disorders (SCARED) (Birmaher et al., 1997), a scale that measures anxiety symptoms in pediatric population with demonstrated internal consistency, construct, and discriminant validity (Birmaher et al., 1999; Monga et al., 2000).

Exclusion criteria were: pervasive developmental disorders, Tourette's syndrome, lifetime history of psychosis, neurological disorder, history of head trauma, ongoing non-psychiatric medical illness requiring medication that may have psychotropic effects such as *β*-blockers or corticosteroids, and IQ < 80.

### Experimental design

The AST was modified from previous studies (Blair et al., 2007; Hwang et al., 2016); see the online Supplementary Fig. S1. In each trial, participants saw a central fixation point (400 ms), a positive, neutral, or negative image (400 ms), either a numerical array on *congruent/incongruent* trials, or a blank screen on *view* trials (400 ms), the same image previously displayed (400 ms), and a second blank screen (1300 ms). For congruent/incongruent trials, participants were required to press a button corresponding to how many numbers were displayed (numerosity: 3–6). On *congruent* trials, numerosity matched the *actual number values displayed* (e.g. three 3s). On *incongruent* trials, numerosity *did not match* the number values displayed (e.g. four 3s or six 5s). The numerical gap between numerosity and the number values ranged between 1 (e.g. four 3s) and 3 (e.g. six 3s). Participants were free to respond at any time between the initial numerical presentation and the end of the blank screen display (response window: 1700 ms). Participants were not asked to make a response for view trials.

The images consisted of 16 positive, 16 negative, and 16 neutral pictures selected from the International Affective Picture System (Lang, B., & Cuthbert, 2005). Participants completed two 8 min 16 s long runs. Each run involved 288 trials [32 in each of the nine categories (3 image × 3 condition)] and 96 fixation trials (each of 2500 ms length to generate a baseline and provide additional jitter between trials). Trial order was randomized across participants.

### MRI parameters

Participants were scanned using a 3.0-Tesla Siemens Skyra MRI scanner. Following sagittal localization, functional T2* weighted images were acquired using an echo-planar single-shot gradient *t* echo pulse sequence [repetition time (TR) = 2500 ms, echo time (TE) = 27 ms, flip angle 90^o^, field-of-view (FOV) = 240 mm, 94 × 94 matrix, 2.6 × 2.6 × 2.5 mm voxels]. Images were acquired in 43 slices of 2.5 mm per brain volume (distance factor 21%), with each run lasting 8 min 16 s. In the same session, a high-resolution T1-weighted anatomical image was acquired to aid with spatial normalization [TR = 2200 ms, TE = 2.48 ms, flip angle = 8°; FOV = 230 mm (0.9 × 0.9 × 0.1 mm^3^ voxels), 176 axial slices, 256 × 208 matrix, thickness = 1.0 mm, distance factor 50%] in register with the echo-planar imaging (EPI) data set was obtained covering the whole brain.

### Imaging data preprocessing

Imaging data were preprocessed and analyzed in Analysis of Functional Neuroimages (AFNI) (Cox, 1996). Both individual- and group-level analyses were conducted. At the individual level, functional images from the first four repetitions, collected prior to equilibrium magnetization, were discarded. The participants' anatomical scans were then individually registered to the Talairach and Tournoux atlas (Talairach & Tournoux, 1988). The individuals' functional EPI data were then registered to their Talairach anatomical scan. The EPI data sets for each participant were spatially smoothed (isotropic 6 mm^3^ Gaussian kernel) to reduce variability among individuals and generate group maps. Next, the time series data were normalized by dividing the signal intensity of a voxel at each time point by the mean signal intensity of that voxel for each run and multiplying the result by 100, producing regression coefficients representing percent-signal change. Every TR on which motion exceeded 1 mm was censored.

A model was generated using six motion regressors and the following 10 regressors: negative congruent, negative incongruent, negative view, neutral congruent, neutral incongruent, neutral view, positive congruent, positive incongruent, positive view, and incorrect responses. GLM fitting was performed with these 10 regressors, six motion regressors, and a regressor modeling baseline drift. All regressors were convolved with a canonical hemodynamic response function to account for the slow hemodynamic response (with time point commencing at the time of first image onset). This produced a *β* coefficient and associated *t* statistic for each voxel and regressor. There was no significant regressor collinearity.

### Statistical analyses

To reduce skewness and kurtosis, a Blom transformation was applied to participants' ARI and SCARED scores [Pre/Post-transformation skewness and kurtosis scores: ARI(Pre): 1.335 and 1.403; SCARED(Pre): 1.196 and 0.819; ARI(Post): 0.358 and −0.568; SCARED(Post): 0.195 and −0.158] (Blom, 1958).

#### Clinical correlations

Correlation analyses were conducted to determine the associations between Blom transformed ARI and SCARED scores, age, IQ, sex, whether the individual received a particular diagnosis or not (scored 1 or 0, respectively) and current medication status.

#### Behavioral data

Two 2 (condition: congruent, incongruent) by 3 (emotion: positive, negative, neutral) analyses of covariance (ANCOVAs) were conducted on the accuracy and reaction time (RT), respectively, with Blom-transformed ARI and SCARED scores and IQ as variables.

#### MRI data

Our hypotheses were tested by a full 3 (condition: congruent, incongruent, view) by 3 (emotion: positive, negative, neutral) repeated-measures ANCOVA performed on the BOLD response data which included ARI, SCARED, and IQ scores as mean-centered variables via 3dMVM. Potential core interactions with respect to our hypotheses were: (i) relevant to Hypothesis 1A: emotion-by-ARI, emotion-by-SCARED, emotion-by-ARI-by-SCARED; (ii) relevant to Hypothesis 1B and 2, condition-by-ARI, condition-by-SCARED, condition-by-ARI-by-SCARED; (iii) relevant to Hypothesis 3, condition-by-emotion-by-ARI, condition-by-emotion-by-SCARED, condition-by-emotion-by-ARI-by-SCARED.

Correction for multiple comparisons was performed using a spatial clustering operation in AFNI's 3dClustSim utilizing the autocorrelation function (-acf) with 10 000 Monte Carlo simulations for a whole-brain grey matter mask with an empirical blur of 9.889. The initial threshold was set at *p* = 0.001 (Cox, Chen, Glen, Reynolds, & Taylor, 2017*a*, 2017*b*). This procedure yielded an extant threshold of *k* = 27 voxels, which then results in a cluster-level false-positive probability of *p* < 0.05, corrected for multiple comparisons. To facilitate future meta-analytic work, effect sizes (Partial *η*^2^) for all clusters are reported.

Interactions of ARI/SCARED scores with variables identified via the ANCOVAs were interpreted via correlational analyses using SPSS 22.0 (*p* < 0.05). For four-way interactions, a bootstrapping procedure using the PROCESS macro for SPSS (Preacher & Hayes, 2004) was used to examine how irritability moderated the association of anxiety with BOLD response (mean percent signal change across voxels per condition) within each of the nine emotion-by-condition trial types (*p* = 0.05, Bonferroni corrected). The Johnson–Neyman technique was used to investigate the heterogeneity of the relationship between anxiety level (SCARED scores) and BOLD responses at different levels of irritability level (ARI scores) (Kowalski, Schneiderman, & Willis, 1994). This technique identified specific ranges of irritability (ARI scores) where the relationship between anxiety level (SCARED scores) and BOLD responses was significant (Kowalski et al., 1994).

## Results

### Clinical characteristics

There was a significant positive correlation between: (i) the ARI and SCARED scores (*r* = 0.429, *p* < 0.001); (ii) both and IQ (*r* = −0.228 and 0.218, *p* = 0.004 and *p* = 0.006, respectively); (iii) SCARED scores and sex [*r* = −0.265, *p* = 0.001; SCARED scores were higher in females but not ARI scores and sex (*r* = 0.003, *p* = 0.973)]; and (iv) neither and age (*r* = 0.142 and −0.014, *p* = 0.078 and *p* = 0.865, respectively). Collinearity analyses revealed a lack of significant multicollinearity; variance inflation factor <1.4 in all cases. There were significant positive correlations between ARI scores and *all* seven psychiatric diagnoses assessed and significant positive correlations between SCARED scores and all three anxiety diagnoses and MDD diagnosis (see Table 1). There were positive correlations between ARI and SCARED scores and stimulant usage and between ARI scores and antipsychotic medication usage.

### Behavioral and movement data

#### Accuracy

There was a trend effect of condition [*F*_(1,151)_ = 2750, *p* = 0.099; participants were more accurate for congruent (85.1%, s.d. = 12.4) than incongruent trials (mean = 79.2%, s.d. = 16.2)].

#### Reaction time

There was a significant main effect of condition [*F*_(1,150)_ = 13.54, *p* < 0.001; RT was significantly slower for the incongruent trials (mean = 852.68 s, s.d. = 194.96) than congruent trials (mean = 782.81 s, s.d. = 194.96)].

There were no other significant main effects or interactions (including with ARI/SCARED scores) for either accuracy or RT.

#### Movement

There was no significant relationship between either ARI or SCARED scores and motion in the scanner, as measured by average motion per TR (*r* = −0.005, *p* = 0.957; *r* = −0.090, *p* = 0.339, respectively) or maximum displacement (*r* = 0.052, *p* = 0.580; *r* = 0.040, *p* = 0.666, respectively).

### MRI data

With respect to our hypotheses, regions showed significant condition-by-ARI, condition-by-emotion-by-SCARED, condition-by-emotion-by-ARI-by-SCARED interactions. These are described below. Main effects and all other interactions revealing significant activation within regions are reported in the supplemental materials (online Supplementary Table S1).

### Condition-by-ARI score interaction

**Fig. 2.**
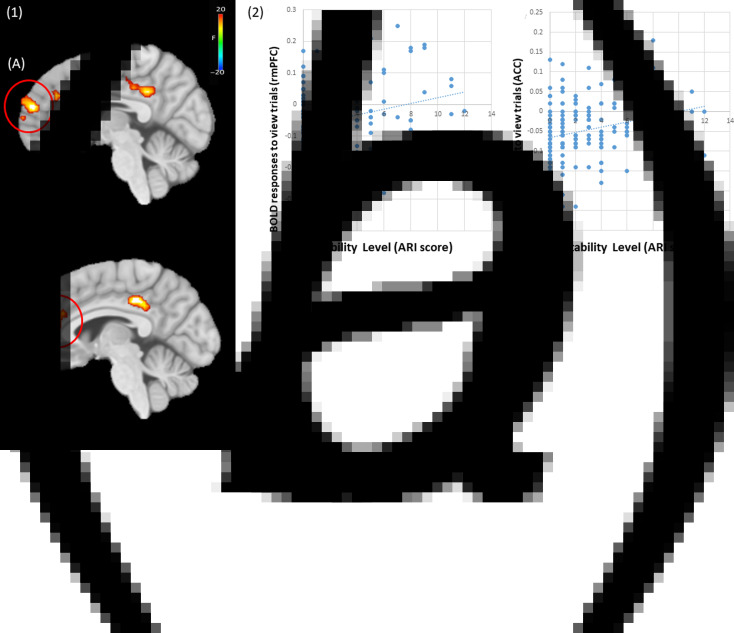
(1) (*a*) Rostro-medial prefrontal cortex (rmPFC; coordinates: 4.5, 52.5, 17.5) and (*b*) anterior cingulate cortex (ACC; coordinates: −4.5, 28.5, 23.5) showing a significant condition-by-irritability symptom level interaction. (2) In both areas, irritability symptom level showed a significantly positive correlation with BOLD responses to view trials.

Regions showing a *condition-by-ARI score interaction* included right rmPFC, left anterior cingulate cortex, and left posterior cingulate cortex; see Table 2. Within all these areas, irritability level was *positively* correlated with BOLD responses to view relative to both congruent (i.e. view-congruent; *r* = 0.469, *p* < 0.001; *r* = 0.455, *p* < 0.001; *r* = 0.318, *p* < 0.001, respectively) and incongruent trials (i.e. view-incongruent; *r* = 0.271, *p* < 0.001; *r* = 0.223, *p* = 0.005; *r* = 0.300, *p* < 0.001, respectively). Notably, within both the right rmPFC and left anterior cingulate cortices, this reflected significant positive correlations between ARI scores and responsiveness to view (*r* = 0.246 and 0.243, *p* = 0.002 and *p* = 0.002, respectively) but not incongruent or congruent trials (*r* = −0.076 and −0.031, *p* = 0.348 and *p* = 0.688, respectively); see Fig. 2. However, within the left posterior cingulate cortex, this reflected *negative* correlations between ARI scores and responsiveness to incongruent and congruent trials (*r* = −0.174, *p* = 0.030; *r* = −0.344, *p* < 0.001, respectively) but not view trials (*r* = 0.092, *p* = 0.254).

**Table 2.** Brain regions showing significant interactions

	Coordinates of peak activation^a^							
Region^b^	Left/right	BA	*x*	*y*	*z*	*F*	Voxels	*η*^2^
Condition-by-irritability symptom level								
Rostro-medial frontal gyrus	Right	9	4.5	52.5	17.5	13.98	84	0.120
Anterior cingulate cortex	Left	32	−4.5	28.5	23.5	10.60	27	0.110
Posterior cingulate cortex	Left	23	−4.5	−28.5	33.5	14.68	116	0.187
Condition-by-emotion-by-anxiety symptom level								
Cuneus	Left	7	−13.5	−76.5	35.5	8.15	36	0.038
Condition-by-emotion-by-irritability-by-anxiety symptom level								
Anterior cingulate cortex	Left	24	−7.5	28.5	11.5	6.68	59	0.027

a Based on the Tournoux and Talairach standard brain template.

b According to the Talairach Daemon Atlas (http://www.nitc.org/projects/tal-daemon/).

### Condition-by-emotion-by-SCARED score interaction

**Fig. 3.**
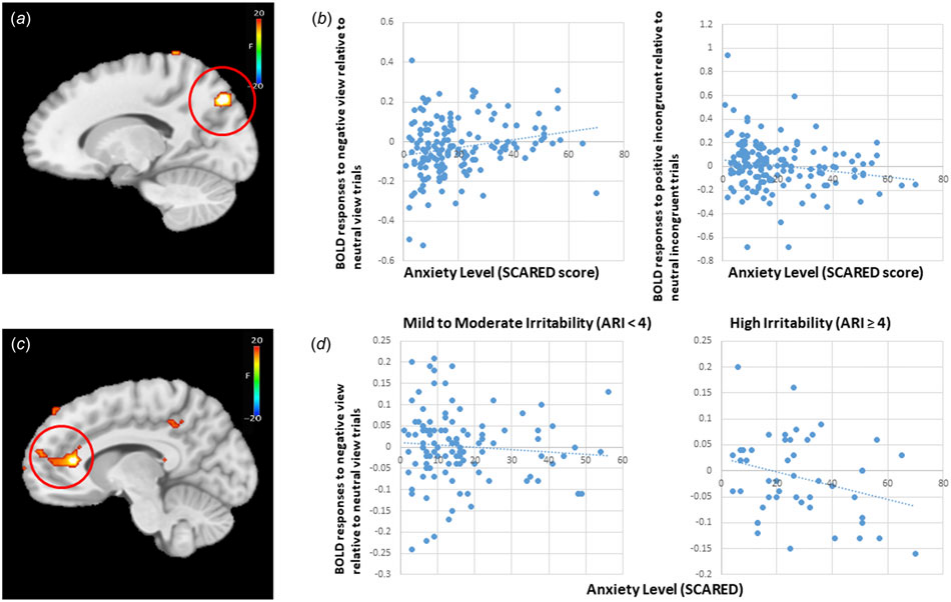
(*a*) Cuneus (coordinates: −13.5, −76.5, 35.5) showing a significant condition-by-emotion-by-anxiety symptom level interaction. (*b*) In this area, anxiety symptom level (SCARED) score showed a significantly positive correlation with the BOLD response to negative view relative neutral view trials, and a significant negative correlation with the BOLD responses to positive incongruent relative to neutral incongruent trials. (*c*) Anterior cingulate cortex (coordinates: −7.5, 28.5, 11.5) showing a significant condition-by-emotion-by-anxiety symptom level-by-irritability symptom level interaction. (*d*) In this area, higher anxiety symptom level predicted decreased BOLD responses to negative view trials relative to neutral view trials, only with the presence of relatively high symptom level of irritability (ARI ⩾ 4; *r* = −0.306, *p* = 0.034). This was not observed for mild to moderate levels of irritability (ARI < 4; *r* = −0.081, *p* = 0.405).

A portion of the left cuneus regions showed a significant *condition-by-emotion-by-SCARED score interaction* (Table 2). Within this region, SCARED scores were negatively correlated with differential response to positive incongruent relative to neutral incongruent trials (i.e. positive incongruent–neutral incongruent; *r* = −0.175, *p* = 0.030) and positively correlated with BOLD responses to negative view relative to neutral view trials (i.e. negative view–neutral view; *r* = 0.207, *p* = 0.010); see Fig. 3.

### Condition-by-emotion-by-ARI score-by-SCARED score interaction

A portion of the left ventral anterior cingulate cortex showed a *condition-by-emotion-by-ARI score-by-SCARED score interaction*; see Table 2. Via the PROCESS macro (Hayes, 2013), we determined that a core component of this interaction reflects a differential response to negative view relative to neutral view trials. Anxiety level was significantly associated with a decreased differential BOLD response to negative view relative to neutral view trials but only in the presence of relatively high irritability (ARI ⩾ 4; *r* = −0.306, *p* = 0.034). There was no significant relationship between anxiety and differential BOLD response at low levels of irritability (ARI < 4; *r* = −0.081, *p* = 0.405); see Fig. 3.

### Follow-up analysis: a group-based approach

We examined the extent to which our ANCOVA irritability results would replicate if we adopted a more conventional group-based approach. Following previous work (Stoddard et al., 2015; Wiggins et al., 2016), this involved contrasting participants with irritability (ARI score 4 or greater) and comparison adolescents (ARI score = 0). As can be seen in online Supplementary Fig. S3 and Table S2, this revealed group-by-condition interactions within the regions of rmPFC, anterior cingulate, and posterior cingulate cortices that were proximal to those identified by the condition-by-ARI interaction in the main analysis.

### Follow-up analyses: potential confounds

We examined potential confounds of medication status via three ANCOVAs excluding participants on stimulants, selective serotonin reuptake inhibitors (SSRIs) and/or antipsychotic medications. In all cases, these exclusions were associated with only minor changes to the results reported in Table 2. For a full overview, see the online Supplementary Tables S3–S5.

Given the significant association between sex and SCARED scores, sex was a potential confound for SCARED-associated findings. To mitigate this concern, BOLD response averages across the regions identified as showing a condition-by-emotion-by-SCARED interaction (left cuneus) or a condition-by-emotion-by-ARI score-by-SCARED interaction (left ventral anterior cingulate cortex) were interrogated using full ANCOVAs that also included sex as a group variable. Within these analyses, left cuneus continued to show a condition-by-emotion-by-SCARED interaction [*F*_(4,556)_ = 6.20; *p* < 0.001] and left ventral anterior cingulate cortex continued to show a condition-by-emotion-by-ARI score-by-SCARED interaction [*F*_(4,556)_ = 5.23; *p* < 0.001]. Within neither region were there significant interactions of sex with task variables.

## Discussion

In this study, we investigated the impairment of neural regions implicated in emotional responding/emotion regulation as a function of participants' levels of irritability and anxiety. There were three main findings: First, irritability (though not anxiety) was *positively* correlated with the BOLD responses during view relative to congruent/incongruent trials within the rmPFC, anterior cingulate, and posterior cingulate cortices. Second, anxiety symptom level (but not irritability) was positively correlated with the BOLD responses to negative emotional stimuli relative to neutral emotional stimuli in the cuneus. In this area, anxiety symptom level was also negatively correlated with the BOLD responses to positive incongruent trials relative to neutral incongruent trials. Third, levels of irritability and anxiety showed an interactive relationship with neural responsiveness in the ventral anterior cingulate cortex as a function of condition-by-emotion. Anxiety level was inversely related to differential responsiveness to negative relative to neutral view trials but only in the presence of significant irritability (ARI score >4).

Our first hypothesis predicted that ARI and/or SCARED scores would be positively associated with responsiveness to distracters – either those with negative valence (Hypothesis 1A) or generally (Hypothesis 1B). With respect to irritability, our results indicated support for Hypothesis 1B. Specifically, the level of irritability was positively correlated with differential BOLD responses to view relative to congruent/incongruent trials within the right rmPFC, left anterior cingulate cortex, and left posterior cingulate cortex. Notably, for the right rmPFC and left anterior cingulate cortex, this reflected a positive association between ARI scores and responsiveness during *view* trials (see Fig. 2). Specifically, increasing irritability was associated with increasing responsiveness during view trials (conditions where automatic regulation, as a function of goal-directed functioning, was not occurring; see also online Supplementary Table S2 for regions showing view>congruent/incongruent activity for the whole sample).

There are several features to note regarding this result. First, atypical responsiveness within these regions to provocation has been noted in previous studies of individuals with irritability (Perlman et al., 2015; Tseng et al., 2018) and during reactive aggression (Lotze, Veit, Anders, & Birbaumer, 2007). Moreover, increased levels of irritability have been associated with atypical connectivity between the amygdala and a proximal region of rmPFC (Stoddard et al., 2017). Irritability has also been associated with structural abnormalities within proximal regions (Besteher et al., 2017). Second, while amygdala and vmPFC/rmPFC showed differential responsiveness to emotional relative to neutral and view relative to congruent/incongruent trials (view>congruent/incongruent; see online Supplementary Fig. S1) in the previous studies, the relationship with ARI score in this study was selective. Increased irritability was *not* associated with increased responsiveness to emotional relative to neutral stimuli. Instead, increased irritability was associated with increased responding within rmPFC to visual stimuli generally during view trials (this was also seen within vmPFC at *p* < 0.01 though not within the amygdala at even more lenient thresholds). Third, rmPFC/vmPFC has been implicated in assessing the salience and relevance of stimuli (Wager et al., 2009; Waugh, Hamilton, Chen, Joormann, & Gotlib, 2012; Waugh, Lemus, & Gotlib, 2014). The suggestion has been made that the intensity of an emotional experience is due in part to the role of these cortical midline structures in affect-based self-referential processing (De Pisapia, Barchiesi, Jovicich, & Cattaneo, 2018; Waugh, Hamilton, & Gotlib, 2010) with rmPFC being particularly implicated in the maintenance of this emotional response (Waugh et al., 2014). We speculate that the current findings reflect a heightened emotional response, reflecting self-referential processing and emotional maintenance that is relatively valence-insensitive, in individuals with higher irritability. For reasons that are unclear, this was not also seen within the amygdala.

There was also evidence of neural hyper-responsiveness as a function of the level of anxiety score (SCARED) but this was specific for negative stimuli and only seen under view conditions within the left cuneus. Previous work has shown that patients with anxiety disorders show increased responsiveness within this area during the allocation of attention to emotional stimuli (Peters, Burkhouse, Kinney, & Phan, 2018; Yoon, Shim, Kim, & Lee, 2016), particularly those with negative valence (Rahko et al., 2010; Sander et al., 2005). Moreover, this region showed abnormalities in patients with anxiety in structural imaging studies (Strawn et al., 2015). However, in contrast to considerable previous works (Blair et al., 2012; Fitzgerald et al., 2017; Salum et al., 2013; Williams et al., 2015), anxiety did not relate to increased amygdala responses to negative stimuli The reasons for this lack are unclear though they may reflect either quality of the paradigm (Blair et al., 2012; Blair et al., 2013) or the unreliability of amygdala activation (van den Bulk et al., 2013).

Our second prediction regarding the association between increased levels of anxiety and reduced recruitment of regions implicated in top-down attentional control during *congruent/incongruent* trials was again partly confirmed within a region of cuneus. This region is adjacent to the parietal cortex and, according to connectivity studies, is part of the dorsal attention network (Fox, Corbetta, Snyder, Vincent, & Raichle, 2006). There have been speculations that it may be involved in very early spatial attention computations (Vossel, Geng, & Fink, 2014). Notably, our finding was proximal to areas showing decreased recruitment in adults with anxiety disorders on the same paradigm (Blair et al., 2012; Blair et al., 2013; White et al., 2015). It should be noted that the reduced recruitment was also specific for incongruent trials in the presence of *positive* rather negative distracters. The reasons for this remain unclear although previous work has also revealed relationships with post-traumatic stress disorder severity that was more marked for positive image trials (White et al., 2015).

Regarding our third prediction on decreased recruitment of regions implicated in emotion regulation as a function of interaction between irritability and anxiety, we observed a distinctive atypical response within ventral anterior cingulate cortex as a product of a condition-by-emotion-by-ARI-by-SCARED interaction. Notably, increased anxiety symptoms were correlated with reduced recruitment to negative relative to neutral *view* trials in youths *only* with significant irritability (ARI ⩾ 4). This region has long been implicated in the processing of emotional stimuli (Bush, Luu, & Posner, 2000; Chiu, Holmes, & Pizzagalli, 2008; Feroz, Leicht, Steinmann, Andreou, & Mulert, 2017) and is specifically implicated in a particular form of emotional-conflict-based emotion regulation (Gyurak et al., 2011). It is possible that increased anxiety in those youths with significant irritability reflects dysfunction in this form of emotion regulation operated by ventral anterior cingulate cortex/ventromedial frontal cortex (Etkin, Buchel, & Gross, 2016). However, it is important to note that the current study did not formally assess emotional-conflict-based emotion regulation. Again, future study is warranted to address this question.

Our finding of increased ventral anterior cingulate cortex activity, a region implicated in emotion regulation, may provide a next step for future clinical trials and treatment. Given that anxiety and irritability are common comorbidities in the pediatric populations (Stoddard et al., 2014), and the co-occurrence of irritability and anxiety is related to distinctive and more severe impairment in the ventral anterior cingulate cortex (Kircanski et al., 2018; Stoddard et al., 2017), treatment options and clinical trials specifically targeting emotion regulation should be considered for this challenging population of youths with comorbid anxiety and irritability.

Irritability and anxiety are common comorbidities in pediatric patients (Stoddard et al., 2014). Pathophysiological models of both show considerable overlap in hypothesizing heightened threat responsiveness in emotion-relevant brain regions (Blair, 2018; Blair et al., 2008; Brotman, Kircanski, Stringaris, Pine, & Leibenluft, 2017; LeDoux & Pine, 2016; Leibenluft, 2017; Salum et al., 2013). However, irritability and anxiety are not inevitably co-morbid. Instead, the current study joins a small body of previous work indicating distinction in their pathophysiology (Kircanski et al., 2018; Stoddard et al., 2017). Like Kircanski et al. (2018) we found that increasing irritability was associated with increased BOLD responses within rmPFC/vmPFC. Moreover, the region of rmPFC implicated in the current study was highly proximal to that showing increased responsiveness to rigged feedback relative to positive feedback in a frustration task as a function of irritability (Tseng et al., 2018). In addition, the region of anterior cingulate cortex showing an irritability-by-anxiety interaction in the current study was proximal to a region showing an irritability-by-anxiety interaction with respect to amygdala connectivity in the study by Stoddard et al. (2017). However, there is still a need for future work to identify which findings are robust, and to clarify the pathophysiology of both irritability and anxiety. Such knowledge will allow interventions to be more targeted toward specific forms of pathophysiology associated with specific symptom profiles.

Six caveats should be considered. First, while this study involves a cross-sectional examination of youths with a wide range of psychiatric diagnoses with respect to internalizing and externalizing conditions, the results only apply to irritability/anxiety in disorders that are well sampled in this study. It may not be applicable to generalize the implications of the current results regarding irritability to other patient populations. Second, we did not implement a structured or semi-structured diagnostic interview. However, even if there was concern about the reliability of the psychiatric diagnoses, it is important to note that the goal of this work was to investigate neural signatures of psychopathologies (irritability and anxiety) *across* various psychiatric diagnoses (Cuthbert & Insel, 2013). Third, many of the youths in the current study had been prescribed psychotropic medications (particularly stimulants and SSRIs; see Table 1). Accordingly, the current results might reflect medication usage rather than irritability/anxiety. However, subsequent analyses excluding the subjects on psychotropic medications yielded similar results to the main analysis (see the online Supplementary Tables S2–4). Fourth, the measure of anxiety, SCARED, is a general index of a variety of different forms of anxiety (generalized and social anxiety, panic school avoidance). These forms of anxiety do not share an identical neural substrate. As such, findings for the SCARED and interactions of the SCARED with irritability might be stronger for particular forms of anxiety. Fifth, SCARED scores were significantly higher in female participants. As such, sex was a confound factor for the interactions involving anxiety. However, it should be noted that follow-up ANCOVAs for BOLD responses within SPSS still revealed the SCARED-based interactions (*p* < 0.001) and neither revealed significant task variable interactions with sex. Future work will determine whether relationships between neural functioning and SCARED scores are moderated by sex. Sixth, our dimensional analyses were conducted using transformed ARI and SCARED scores to reduce the possibility that there would be a disproportionate influence of data points in the tail and/or extreme outliers. However, it is possible that this analysis strategy fails to fully identify the pathophysiology underpinning irritability if, for example, the spread of raw ARI scores more accurately captures this pathophysiology than our transformed scores. Future work may need larger samples and a more aggressive stratification of the original score data set to reduce/remove skewness without having to rely on transformations.

## Conclusion

In conclusion, we observed that irritability was positively associated with responses to visual stimuli during view trials within the right rmPFC and left anterior cingulate cortex, regions implicated in assessing the salience and relevance of emotional stimuli and maintaining the emotional response to these stimuli. As such, atypical maintenance of emotion within rmPFC may exacerbate the difficulties faced by youths with irritability (cf. Leibenluft, Blair, Charney, and Pine, 2003). The current data also raise the possibility that increased anxiety in youths with significant irritability disrupts an automatic emotional conflict-based form of emotion regulation that is particularly associated with the ventral anterior cingulate cortex/vmPFC (Etkin et al., 2016). However, this latter possibility requires additional, future empirical work.

## Supplementary material

For supplementary material accompanying this paper visit https://doi.org/10.1017/S0033291720001397. click here to view supplementary material
