# Supplementary material for: Interaction of irritability and anxiety on emotional responding and emotion regulation: a functional MRI study
Source: Psychol Med. 2020 Jun 25;51(16):2778–88. doi: 10.1017/S0033291720001397 (PMC7759590; doi:10.1017/S0033291720001397)
Supplement: Supplementary file 1 [file S0033291720001397sup001.docx]

**Supplementary Material**

**Figure 1: Conceptual overview of the functional processes/neural systems underpinning performance on the Affective Stroop Task (the AST).** Blue arrows show information flow between systems implicated in top down attention. Specifically, on goal-directed congruent and incongruent trials, dorsomedial and dorsolateral frontal and parietal cortices prime the representation of the number stimulus. This increases the representational strength of this stimulus and reduces the strength of the representation of any distracter stimuli via representational competition (cf. Desimone & Duncan, 1995). Red arrows show information flow between systems implicated in emotional responding. Visual image distracters are represented in visual/temporal cortex. They activate the amygdala which, because of reciprocal connections with visual/temporal cortex, increases the strength of the representations of these distracters. While amygdala activation is stronger for emotional distracters it occurs also to neutral distracters on this task because of their salience (i.e., their social component and novelty). The amygdala activates ventromedial frontal cortex (vmFC) and in turn rostro-medial frontal cortex (rmFC).While vmPFC shows greater responding to emotional relative to neutral images (see Suppmental Figure 2), rmFC shows comparable responding to both in the context of view trials. This may represent some form of more general self-referential processing and emotional maintenance to salient stimuli (De Pisapia, Barchiesi, Jovicich, & Cattaneo, 2018; Waugh, Hamilton, & Gotlib, 2010; Waugh, Lemus, & Gotlib, 2014).

**Figure 2: Data from the main effect of Emotion and the main effect of Condition.** The amygdala and vmFC show strong activations to emotional relative to neutral stimuli and view relative to congruent/incongruent trials. In addition, rmFC shows strong activation to view relative to congruent/incongruent trials.

**Figure 3: Data from the analysis contrasting the group of participants with ARI scores > 4 and comparison individuals with ARI scores = 0: The results of the Group-by-Condition interaction.**


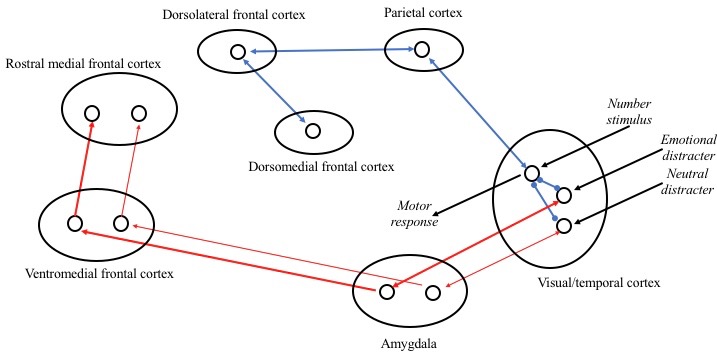
SUPPLEMENTAL FIGURE 1


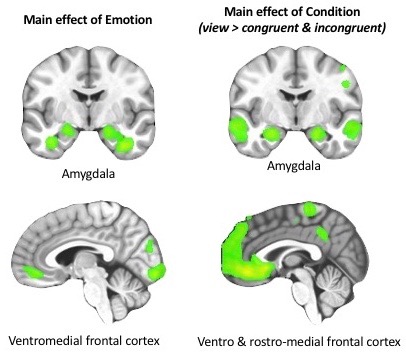
SUPPLEMENTAL FIGURE 2:

**
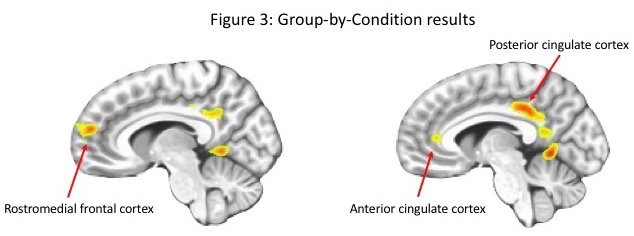
**SUPPLEMENTAL FIGURE 3:

**SUPPLEMENTARY TABLES NFORMATION**

**Tables Index:**

Table S1: Regions showing significant main effect of condition or emotion

Table S2: Regions showing a significant Group-by-Condition interaction

Table S3: Significant areas of activation from the analysis involving antipsychotic use as an additional covariate.

Table S4: Significant areas of activation from the analysis involving SSRI use as an additional covariate.

Table S5: Significant areas of activation from the analysis involving stimulant use as an additional covariate.

Table S1. Brain areas showing main effect of condition and main effect of emotion

|  |  | **Coordinates of Peak Activation** | | | |  |  |  |
| --- | --- | --- | --- | --- | --- | --- | --- | --- |
| **Region^a^** | **Left/Right** | **BA** | **x** | **y** | **z** | **F** | **Voxels** |  |
| **Main effect of condition** |  |  |  |  |  |  |  |  |
| Middle frontal gyrus | Right | 9 | 31.5 | 31.5 | 32.5 | 55.49 | 152 |  |
| Middle frontal gyrus | Left | 9 | -37.5 | 34.5 | 26.5 | 100.00 | 306 |  |
| Inferior frontal gyrus | Right | 45 | 52.5 | 31.5 | 8.5 | 52.03 | 90 |  |
| Inferior frontal gyrus | Left | 47 | -40.5 | 28.5 | -3.5 | 77.70 | 191 |  |
| Inferior parietal lobule | Left | 40 | -37.5 | -35.5 | 64.5 | 100.00 | 12786 |  |
| Anterior cingulate cortex | Left | 24 | -4.5 | 22.5 | -3.5 | 49.96 | 207 |  |
| Posterior cingulate cortex | Right | 23 | 4.5 | -28.5 | 26.5 | 67.71 | 108 |  |
| Precuneus | Left | 39 | -43.5 | -70.5 | 35.5 | 38.80 | 48 |  |
| **Main effect of emotion** |  |  |  |  |  |  |  |  |
| Ventromedial prefrontal cortex | Right | 10 | 4.5 | 40.5 | -6.5 | 20.93 | 39 |  |
| Inferior frontal gyrus | Right | 46 | 49.5 | 28.5 | 14.5 | 25.76 | 94 |  |
| Fusiform gyrus | Right | 20 | 37.5 | -37.5 | -18.5 | 100.00 | 2882 |  |
| Parahippocampal gyrus | Right | 36 | 28.5 | -40.5 | -6.5 | 38.00 | 54 |  |
| Parahippocampal gyrus | Left | 36 | -28.5 | -40.5 | -6.5 | 45.87 | 58 |  |
| Amygdala | Right |  | 19.5 | -4.5 | -12.5 | 31.67 | 37 |  |
|  |  |  |  |  |  |  |  |  |
|  |  |  |  |  |  |  |  |  |

^a^According to the Talairach Daemon Atlas (<http://www.nitrc.org/projects/tal-daemon>).

Table S2. Brain regions showing significant Group-by-Condition interactions.

|  | Coordinates of peak activation^b^ | | | | |  | |  |  |
| --- | --- | --- | --- | --- | --- | --- | --- | --- | --- |
| Region^a^ | Left/Right | BA | x | y | z | Voxels |  |  |  |
| **Group-by-Condition** |  |  |  |  |  |  |  |  |  |
| Rostromedial frontal cortex | Right | 9 | 8 | 50 | 17 | 55 |  |  |  |
| Anterior cingulate cortex | Left | 32 | -7 | 29 | 2 | 27 |  |  |  |
| Superior frontal gyrus | Right | 8 | 20 | 26 | 44 | 116 |  |  |  |
| Inferior parietal lobule | Right | 39 | 41 | -64 | 41 | 82 |  |  |  |
| Culmen extending to posterior cingulate cortex | Left | 30/31 | -4 | -55 | -1 | 292 | |  |  |

^a^According to the Talairach Daemon Atlas (<http://www.nitc.org/projects/tal-daemon/>).

^b^Based on the Tournoux and Talairach standard brain template.

Table S3. Brain regions showing significant interactions from the repeated measures ANCOVA excluding those taking antipsychotic medications. Activations are effects observed in whole brain analyses significant at p<0.001, corrected for multiple comparisons (significant at p<0.05).

|  | Coordinates of peak activation^b^ | | | | |  |  |  | |
| --- | --- | --- | --- | --- | --- | --- | --- | --- | --- |
| Region^a^ | Left/Right | BA | x | y | z | F | Voxels | |  |
| **Condition by irritability symptom level** |  |  |  |  |  |  |  | |  |
| Rostro-medial frontal gyrus | Right | 9 | 14 | 59 | 20 | 11.69 | 62 | |  |
| Anterior cingulate cortex | Left | 32 | -4 | 29 | 23 | 8.63 | 54 | |  |
| Posterior cingulate cortex | Left | 23 | -4 | -28 | 32 | 13.67 | 95 | |  |
| **Condition by emotion by anxiety symptom level** |  |  |  |  |  |  |  | |  |
| Cuneus* | Left | 7 | -10 | -76 | 35 | 7.59 | 48 | |  |
| **Condition by emotion by irritability by**  **anxiety symptom level** |  |  |  |  |  |  |  | |  |
| Anterior cingulate cortex | Left | 24 | -7 | 29 | 11 | 8.11 | 66 | |  |

Table S4. Brain regions showing significant interactions from the repeated measures ANCOVA excluding those taking SSRIs. Activations are effects observed in whole brain analyses significant at p<0.001, corrected for multiple comparisons (significant at p<0.05).

|  | Coordinates of peak activation^b^ | | | | |  |  |  | |
| --- | --- | --- | --- | --- | --- | --- | --- | --- | --- |
| Region^a^ | Left/Right | BA | x | y | z | F | Voxels | |  |
| **Condition by irritability symptom level** |  |  |  |  |  |  |  | |  |
| Rostro-medial frontal gyrus | Right | 9 | 14 | 59 | 20 | 12.60 | 74 | |  |
| Anterior cingulate cortex | Left/ Right | 32 | 8 | 35 | 26 | 8.14 | 209 | |  |
| Posterior cingulate cortex | Left | 23 | -4 | -28 | 32 | 15.17 | 115 | |  |
| **Condition by emotion by anxiety symptom level** |  |  |  |  |  |  |  | |  |
| Cuneus | Left | 7 | -10 | -76 | 35 | 8.99 | 54 | |  |
| **Condition by emotion by irritability by**  **anxiety symptom level** |  |  |  |  |  |  |  | |  |
| Anterior cingulate cortex | Left | 24 | -7 | 29 | 11 | 6.93 | 50 | |  |

Table S5. Brain regions showing significant interactions from the repeated measures ANCOVA excluding those taking stimulants. Activations are effects observed in whole brain analyses significant at p<0.001, corrected for multiple comparisons (significant at p<0.05). *significant at p<0.005.

|  | Coordinates of peak activation^b^ | | | | |  |  |  | |
| --- | --- | --- | --- | --- | --- | --- | --- | --- | --- |
| Region^a^ | Left/Right | BA | x | y | z | F | Voxels | |  |
| **Condition by irritability symptom level** |  |  |  |  |  |  |  | |  |
| Rostro-medial frontal gyrus | Right | 9 | 11 | 62 | 20 | 10.88 | 40 | |  |
| Anterior cingulate cortex* | Left | 32 | -7 | 29 | 26 | 9.20 | 57 | |  |
| Posterior cingulate cortex | Left | 23/31 | -4 | -31 | 32 | 12.52 | 82 | |  |
| **Condition by emotion by anxiety symptom level** |  |  |  |  |  |  |  | |  |
| Cuneus* | Left | 7 | -13 | -76 | 35 | 7.14 | 44 | |  |
| **Condition by emotion by irritability by**  **anxiety symptom level** |  |  |  |  |  |  |  | |  |
| Anterior cingulate cortex | Left | 24 | -7 | 29 | 11 | 9.47 | 82 | |  |

**REFERENCES**

De Pisapia, N., Barchiesi, G., Jovicich, J., & Cattaneo, L. (2018). The role of medial prefrontal cortex in processing emotional self-referential information: a combined TMS/fMRI study. *Brain Imaging and Behavior,* 13(3):603-614. doi:10.1007/s11682-018-9867-3

Desimone, R., & Duncan, J. (1995). Neural mechanisms of selective visual attention. *Annual Review of Neuroscience, 18*, 193-222. doi: 10.1146/annurev.ne.18.030195.001205

Waugh, C. E., Hamilton, J. P., & Gotlib, I. H. (2010). The neural temporal dynamics of the intensity of emotional experience. *Neuroimage, 49*(2), 1699-1707. doi:10.1016/j.neuroimage.2009.10.006

Waugh, C. E., Lemus, M. G., & Gotlib, I. H. (2014). The role of the medial frontal cortex in the maintenance of emotional states. *Social Cognitive and Affective Neuroscience, 9*(12), 2001-2009. doi:10.1093/scan/nsu011
